# Meiotic analyses show adaptations to maintenance of fertility in X1Y1X2Y2X3Y3X4Y4X5Y5 system of amazon frog *Leptodactylus pentadactylus* (Laurenti, 1768)

**DOI:** 10.1038/s41598-020-72867-x

**Published:** 2020-10-01

**Authors:** Renata Coelho Rodrigues Noronha, Bruno Rafael Ribeiro de Almeida, Marlyson Jeremias Rodrigues da Costa, Cleusa Yoshiko Nagamachi, Cesar Martins, Julio Cesar Pieczarka

**Affiliations:** 1 Laboratório de Citogenética, Centro de Estudos Avançados em Biodiversidade, Instituto de Ciências Biológicas, Universidade Federal do Pará, Campus Guamá, Rua Augusto Corrêa, no. 01, Guamá, Belém, Pará CEP 66075-900 Brazil; 2 Departamento de Biologia Estrutural e Funcional, Instituto de Biociências de Botucatu, Universidade Estadual Paulista (UNESP), Campus Botucatu, Botucatu, São Paulo CEP 18618689 Brazil

**Keywords:** Biological techniques, Cell biology, Developmental biology, Genetics, Environmental sciences

## Abstract

Heterozygous chromosomal rearrangements can result in failures during the meiotic cycle and the apoptosis of germline, making carrier individuals infertile. The Amazon frog *Leptodactylus pentadactylus* has a meiotic multivalent, composed of 12 sex chromosomes. The mechanisms by which this multi-chromosome system maintains fertility in males of this species remain undetermined. In this study we investigated the meiotic behavior of this multivalent to understand how synapse, recombination and epigenetic modifications contribute to maintaining fertility and chromosomal sexual determination in this species. Our sample had 2n = 22, with a ring formed by ten chromosomes in meiosis, indicating a new system of sex determination for this species (X1Y1X2Y2X3Y3X4Y4X5Y5). Synapsis occurs in the homologous terminal portion of the chromosomes, while part of the heterologous interstitial regions performed synaptic adjustment. The multivalent center remains asynaptic until the end of pachytene, with interlocks, gaps and rich-chromatin in histone H2A phosphorylation at serine 139 (γH2AX), suggesting transcriptional silence. In late pachytene, paired regions show repair of double strand-breaks (DSBs) with RAD51 homolog 1 (Rad51). These findings suggest that Rad51 persistence creates positive feedback at the pachytene checkpoint, allowing meiosis I to progress normally. Additionally, histone H3 trimethylation at lysine 27 in the pericentromeric heterochromatin of this anuran can suppress recombination in this region, preventing failed chromosomal segregation. Taken together, these results indicate that these meiotic adaptations are required for maintenance of fertility in *L. pentadactylus*.

## Introduction

During the evolution of vertebrates, the XY and ZW chromosomes developed independently through translocations and fusion/fission rearrangements, generating multiple sex chromosomes^1–3^. In the specific case of reciprocal translocations, sex chromosomes and autosome pairs suffer breaks, and the resulting segments are reciprocally linked between the elements involved, in order to maintain the integrity of the genome, and in this way, rearranged autosomes come to be considered derived sex chromosomes^3,4^. For example, meiosis I in monotremes involves the formation of a chain composed of ten sex chromosomes, X1Y1X2Y2X3Y3X4Y4X5Y5, resulting from multiple translocation^5^. Thus, when perform synapses during meiotic cycles consist of various types of multivalent associations, ranging from simple trivalent to complex forms, involving several karyotype elements^5–7^. These systems have also been observed in other animals, including bats^6–8^, rodents^3^, birds^9^, fish^10^, lizards^11^, beetles^12^, butterflies^13^, termites^14^, spiders^15^ and scorpions^16^.

The evolution of multiple sex chromosomes may involve the accumulation of repetitive sequences, heterochromatinization, deletion, degeneration, and reduction of recombination rates at homologous sequences^17^. These changes can also affect the meiotic pairing in simple or multi-chromosomal sexual systems. In eutherian mammals, for example, the pairing between the X and Y chromosomes occurs only in the small pseudoautosomal region (which maintains homology between both), through the formation of a short synaptonemal complex (SC)^4^. The synaptonemal complex is a protein structure, responsible by synapse of homologous during the zygotene and pachytene. It is formed by two lateral elements, which bind to cohesins present in the loops of homologous chromosomes (structural maintenance of chromosomes protein 3—SMC3—for example), and later are joined by a central element^7,18^. In most eukaryotes, the co-alignment of homologous regions of autosomes or sex chromosomes and the organization of the SC is dependent on the formation of double DNA breaks (DSBs) made by the enzyme Meiotic recombination protein SPO11 (Spo11) at the beginning of meiosis I^19^. Once DSBs are formed, the adjacent chromatin is enriched for histone H2A phosphorylation at serine 139 (γH2AX) to recruit repair factors^18,19^. Then, the Rad51 and Meiotic recombination protein DMC1/LIM15 homolog (Dmc1) recombinases associate with the DSB sites, forming nucleoproteic filaments, and invade the homologous chromosome with the objective of performing the repair of the break by recombination, promoting the necessary physical approximation for formation of the SC^18^.

A high degree of morphological differentiation of multiple sex chromosomes can enhance the numbers of asynaptic regions during pairing of multivalent chromosomes during meiosis I. Genes located in asynaptic regions are transcriptionally inactivated during prophase I through *meiotic silencing of unsynapsed chromatin* (MSUC)^20^. Sex chromosomes of some vertebrates and insects have a specialized form of MSUC called *meiotic sex chromosome inactivation* (MSCI)^6^. In eutherian mammals and marsupials, MSUC usually begins with the insertion of the repair protein BRCA 1 at non-synapsed axial elements^21^. Subsequently, other factors, including the kinase ataxia telangiectasia and Rad3 related (ATR), are recruited and promote formation of γH2AX, which silences autosomal and sex chromosome associated sequences^22,23^. Others epigenetic modifications, such as H3K27me3 and histone H3 tri-methylation at lysine 9 (H3K9me3) are also observed during early phases prophase I and contribute to MSUC^24^. This phenomenon evolved independently in several taxa. During meiosis in the platypus, for example, sex chromosomes are not remodeled by γH2AX^25^. MSUC patterns in birds, such as *Gallus,* differ from those in eutherian mammals, because epigenetic modifications of the W chromosome in birds disperse and inactivate Z chromatin during heterologous synapsis of these two chromosomes^26^. In other vertebrates, this process is not well understood or has not been determined.

Sex chromosomes of some anuran amphibians show male heterogamety (XY), while in other species, heterogamety is observed in females (ZW), the latter being the ancestral condition in the order *Anura*^27^. Others sex chromosome systems are found in these animals, such as 0W/00 in *Leiopelma hochstetteri*^28^. Multiple sex chromosomes have also been observed in anurans, such as the X1X1X2X2/X1X2Y system in *Eleutherodactylus maussi*^29^. Despite this diversity of sex chromosome systems in frogs, less is known about the meiotic behavior of their allosomes. Multivalent systems were observed in species of the genera *Physalaemus*^30^ and *Eleutherodactylus*^31^, but were not associated with sex differences.

The Amazon frog *Leptodactylus pentadactylus* (Anura, Leptodactylidae), which is widely distributed throughout neotropical regions, has the karyotype 2n = 22 and a fundamental number ranging from 42 to 44^32,33^. Meiosis I in this species involves the formation of a multivalent system composed of 12 heteromorphic elements^32^. This heteromorphism occurs only in males, and the presence of microsatellite GATA (specific to the heterochromatin of the Y/W chromosome) in two elements of the meiotic ring suggests that this association constitutes a multi-chromosome sex determination system, X1Y1X2Y2X3Y3X4Y4X5Y5X6Y6^34^. Heterozygous chromosomal rearrangements, as observed in this anuran species, can promote serious damage to the meiotic process, including synapsis, recombination, chromatin remodeling and chromosomal segregation^19^. These failures may be recognized by the pachytene checkpoint during prophase I, activating chain apoptosis in germ lines and reducing the fertility of carriers of individual rearrangements^35^. The present study investigated the meiotic behavior of this multivalent system in males of *L. pentadactylus* by immunocytogenetic analysis to determine the mechanisms by which synapse, recombination and epigenetic modifications contribute to the maintenance of fertility and the determination of sex chromosomes in this species.

## Results

**Figure 1:**
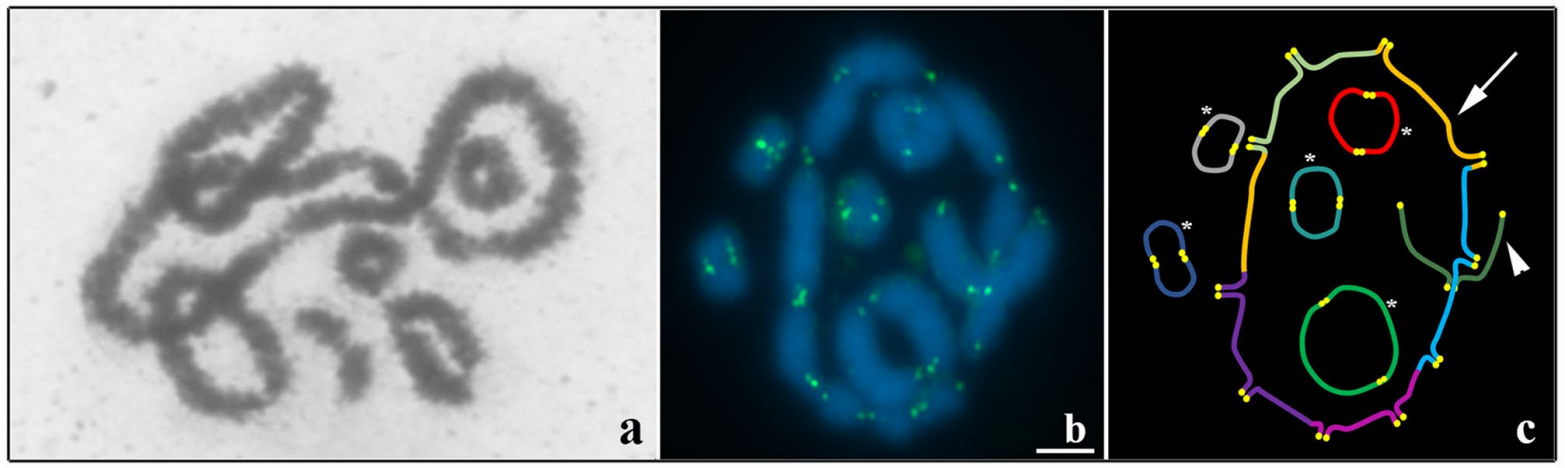
Meiotic multivalent in *L. pentadactylus*. (**a**) Diakinesis showing ring (ten chromosomes) and six regular bivalents. (**b**) FISH with telomeric probe (green) in diakinesis cell of *L. pentadactylus*. (**c**) Schematic representation of cell in (“**b**”); arrow = meiotic ring, arrowheads = bivalent with an interstitial chiasma, asterisks = bivalent with two terminal chiasmas. Barr = 10 μm.

The studied specimens showed a karyotype with 2n = 22 chromosomes. FISH analysis with a telomeric probe was performed in ten diakinesis of each individual and showed marking only on the chromosomal ends of the five bivalents with two terminal chiasms, one bivalent with an interstitial chiasm and a multivalent formed by five chromosomal pairs. No interstitial markings were observed (Fig. 1).

**Figure 2:**
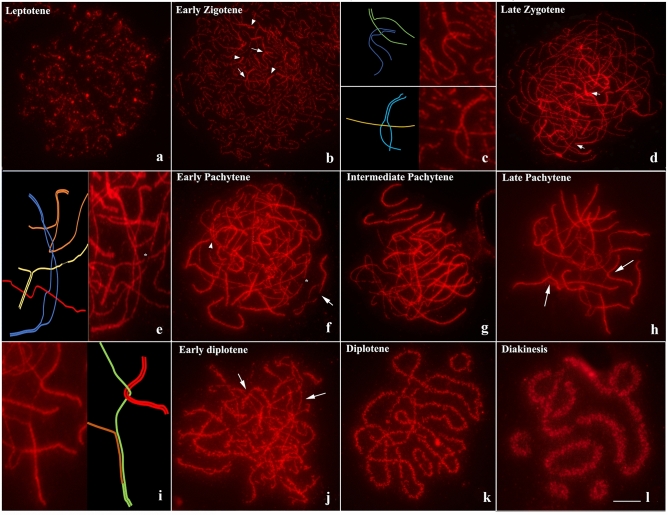
Temporal dynamics of synaptonemal complex in *L. pentadactylus* through SMC3 (red)*.* (**a**) Leptotene. (**b**) Early zygotene; the arrows indicate interlocks, arrowheads show regions of homologous early pairing. (**c**) Schematic representation of interlocks observed in (“**b**”). (**d**) Late zygotene: arrow indicates tips of bivalents realizing synapsis. (**e**) Schematic representation of bivalent evidenced in “d”, highlighting interlocks along of non-synapsed region. (**f**) Early pachytene; asterisk demonstrate gaps, arrowhead shows interlock, and arrow indicates bivalent non-synapsed. (**g**) Intermediate pachytene. (**h**) Late pachytene; the arrows indicate interlocks between bivalent and asynaptic regions of multivalent. (**i**) Schematic representation of non-homologous synapsis and interlock evidenciated in (**h**). (**j**) Early diplotene (arrows show asynaptic regions). (**k**) Late diplotene. (**l**) Diakinesis. Barr = 10 μm.

The meiotic behavior of *L. pentadactylus* chromosomes was investigated by immunodetection of SMC3 that marks the axis of cohesins associated with the complex synaptonemal. Quantitative analysis of the progression of the synapse during the phases of prophase I is shown in Fig. 6a. During leptotene, a short lateral axis of the synaptonemal complex formed and remained asynaptic (Fig. 2a). During early zygotene, homologous pairing started through the chromosomal tips, which were located at a pole of the cell nucleus (Fig. 2b). Formation of interlocks was also observed during this phase (Fig. 2c). In late zygotene, the polarization of chromosomal tips was no longer visible, but interlocks between bivalents and the asynaptic axis, as well as gaps, were observed (Fig. 2d,e).

During pachytene, the synaptonemal complex formed and extended throughout the bivalents; during late pachytene, long asynaptic regions, gaps and interlocks were observed (Fig. 2f,g). Synapsis was incomplete in the center of the multivalent complex, suggesting an absence of homology in this region (Fig. 2h,i). None of the cells in pachytene showed an open configuration of the meiotic ring. The synaptonemal complex became disordered during early diplotene, making it possible to detect asynaptic regions during this phase (Fig. 2j). Five bivalents and the meiotic ring could be distinguished during diplotene and diakinesis (Fig. 2k,l). Moreover, the disorganization of the synaptonemal complex during these phases resulted in the dispersion of SMC3 cohesion throughout the chromosome axis (Fig. 2k,l).

Immunodetection of Synaptonemal Complex Protein 3 (SYCP3), a protein of the lateral element of the synaptonemal complex, demonstrated similar results to SMC3, in relation to the dynamics of pairing, synapse and synaptic adjustment along the prophase I of *L. pentadactylus* (Fig. 7a,d). Immunolocalization of CREST on SYCP3 axes in early pachytene demonstrated that in this stage centromeric region is synapsed in some axes, while in others was observed process of pairing of kinetochores (Fig. 7a–c). In intermediate/late pachytene, immunodetection of CREST revealed a pattern similar to observed in early pachytene, with presence of centromeres in synapsed/adjusted or asynaptic regions of multivalent (Fig. 7d–f).

**Figure 3:**
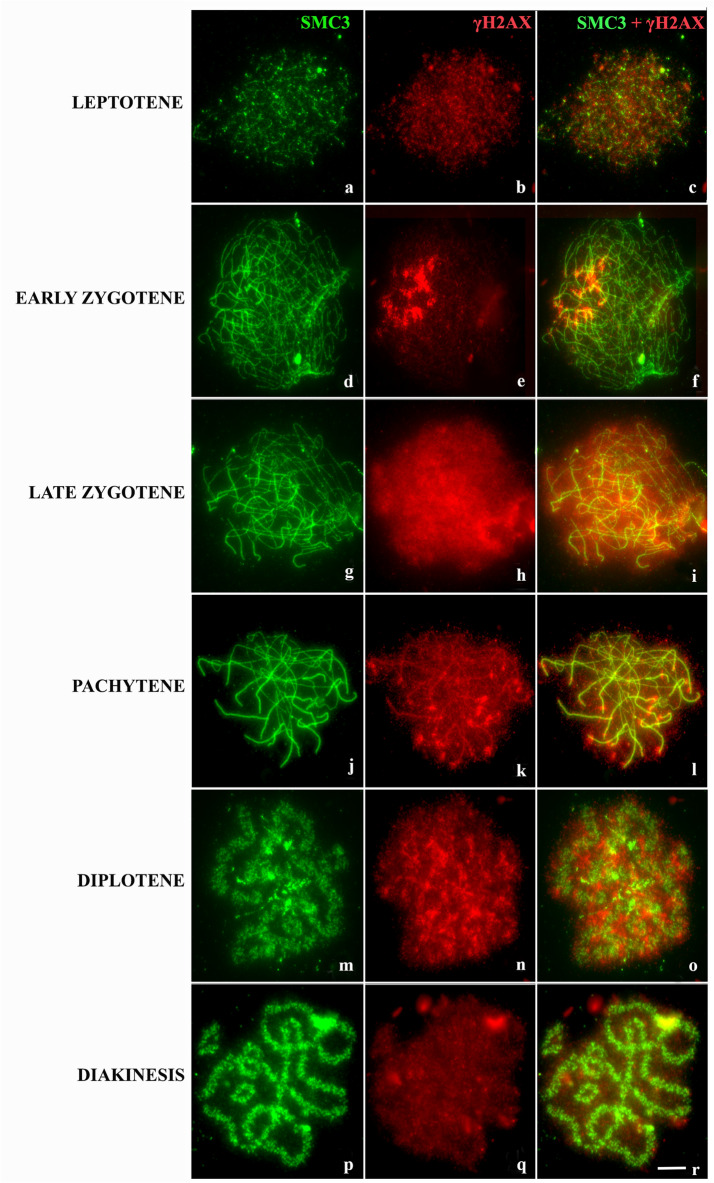
Distribution of γH2AX epigenetic modification along the synaptic process in *L. pentadactylus*. The cohesin SMC3 is shown in green, and γH2AX in red. In the right column, overlay both images. (**a–c**) Leptotene. (**d-f**) Zygotene; note the strongly marked terminal regions. (**g–i**) Late zygotene. (**j–l**) Intermediate/late pachytene. (**m–o**) Diplotene. (**p–r**) Diakinesis. Barr = 10 μm.

To verify the distribution of DSBs performed by Spo11 and the occurrence of MSUC during prophase I of *L. pentadactylus*, we performed immunodetection of γH2AX. Incubation with antibody to γH2AX revealed that this histone variant was diffusely present during leptotene (Fig. 3a–c) but was strongly present at the chromosomal ends, the start of synapses, in zygotene cells (Fig. 3d–f). γH2AX was strongly expressed throughout the chromatin during the transition from zygotene to pachytene (Fig. 3g–i). During intermediate pachytene, this marker was weakly expressed throughout the chromatin, but was strongly expressed in synapsed regions (Fig. 3j–l). During diplotene, γH2AX signals were observed in the chromosomal chain and the bivalent (Fig. 3m–o). γH2AX signals, however, were progressively reduced during diakinesis (Fig. 3p–r).

**Figure 4:**
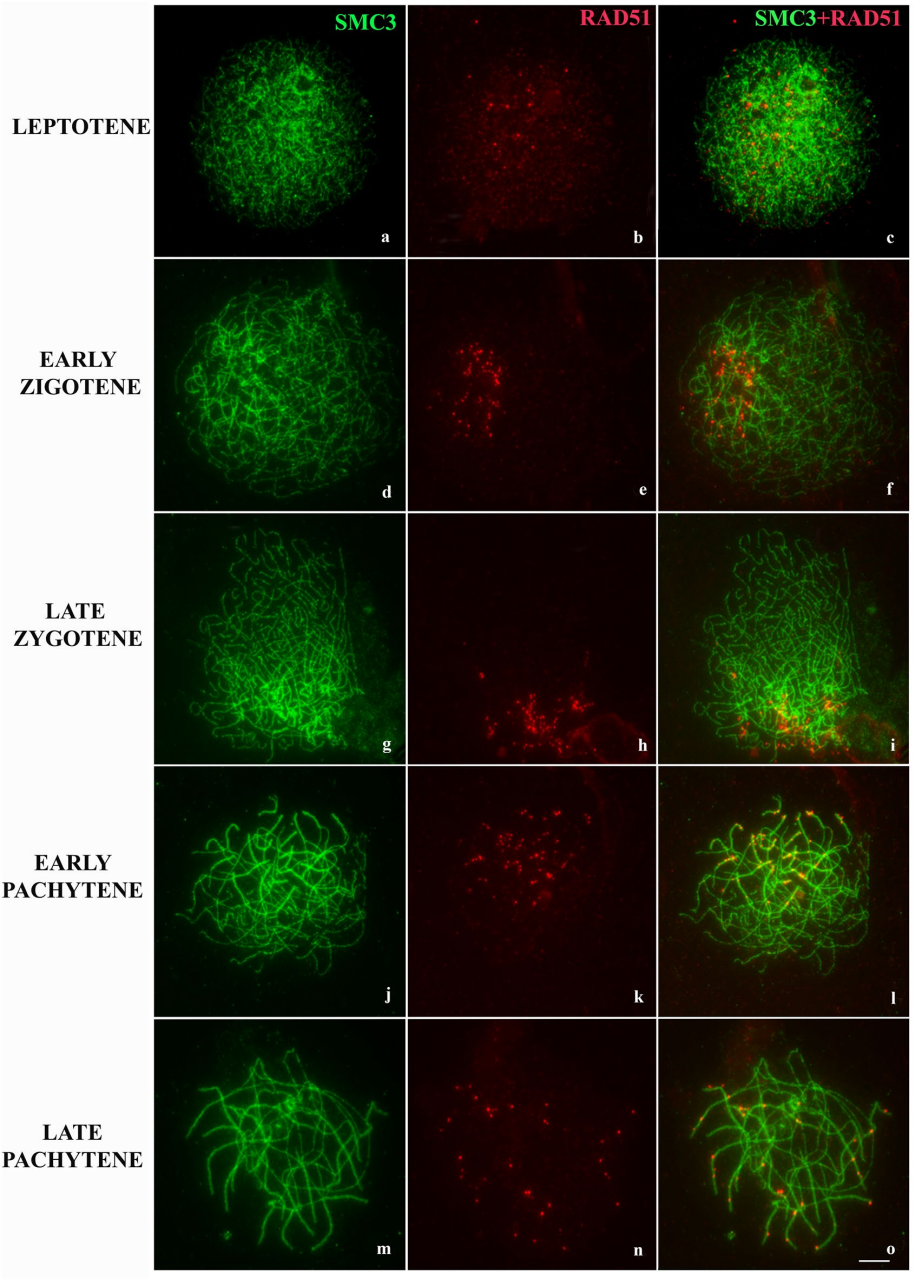
Immunolocation of Rad51 in *L. pentadactylus* prophase I. The cohesin SMC3 is shown in green, and Rad51 in red. In the right column, overlay both images. (**a–c**) Leptotene. (**d–f**) Leptotene/zygotene transition. (**g–i**) Zygotene. (**j–l**) Initial pachytene. (**m–o**) Late Pachytene. Barr = 10 μm.

The repair dynamics of DSBs in the early stages of *L. pentadactylus* prophase I was investigated through the immunodetection of Rad51. This recombinase was slightly expressed during leptotene (Figs. 4a–c, 6b). In early zygotene, Rad51 show polarized expression in the nucleus, predominantly on non-synapsed SMC3 axes in an average 48.88 ± 14.24 (n = 24 cells) (Figs. 4d–f, 6b). During late zygotene, increases in the amount of Rad51 foci were observed on the meiotic axes with synapse started (18.63 ± 8.827, n = 19) (Figs. 4g–i, 6b). In early pachytene, the number foci Rad51 decreases in both synapsed/adjusted (4.905 ± 2.385, n = 21) and asynaptic regions (8.905 ± 3.807, n = 21) of ring and bivalents (Figs. 4j–l, 6b). Expression of Rad51 in intermediate/late pachytene increases in synapsed/adjusted SMC3 axes (8.10 ± 4.040, n = 19) (Fig. 4m–o). ANOVA test demonstrated that the difference in the amount of Rad51 foci in the pachytene is significant compared to the zygotene (p < 0.001). Rad51 expression, however, was not observed during diplotene. Analysis of the colocalization of Rad51 and telomeres showed that this association is more frequent during the early and late zygotene, with a statistically significant decrease during the transition to the pachytene (Kruskal–Wallis p < 0.001) (Fig. 6c).

**Figure 5:**
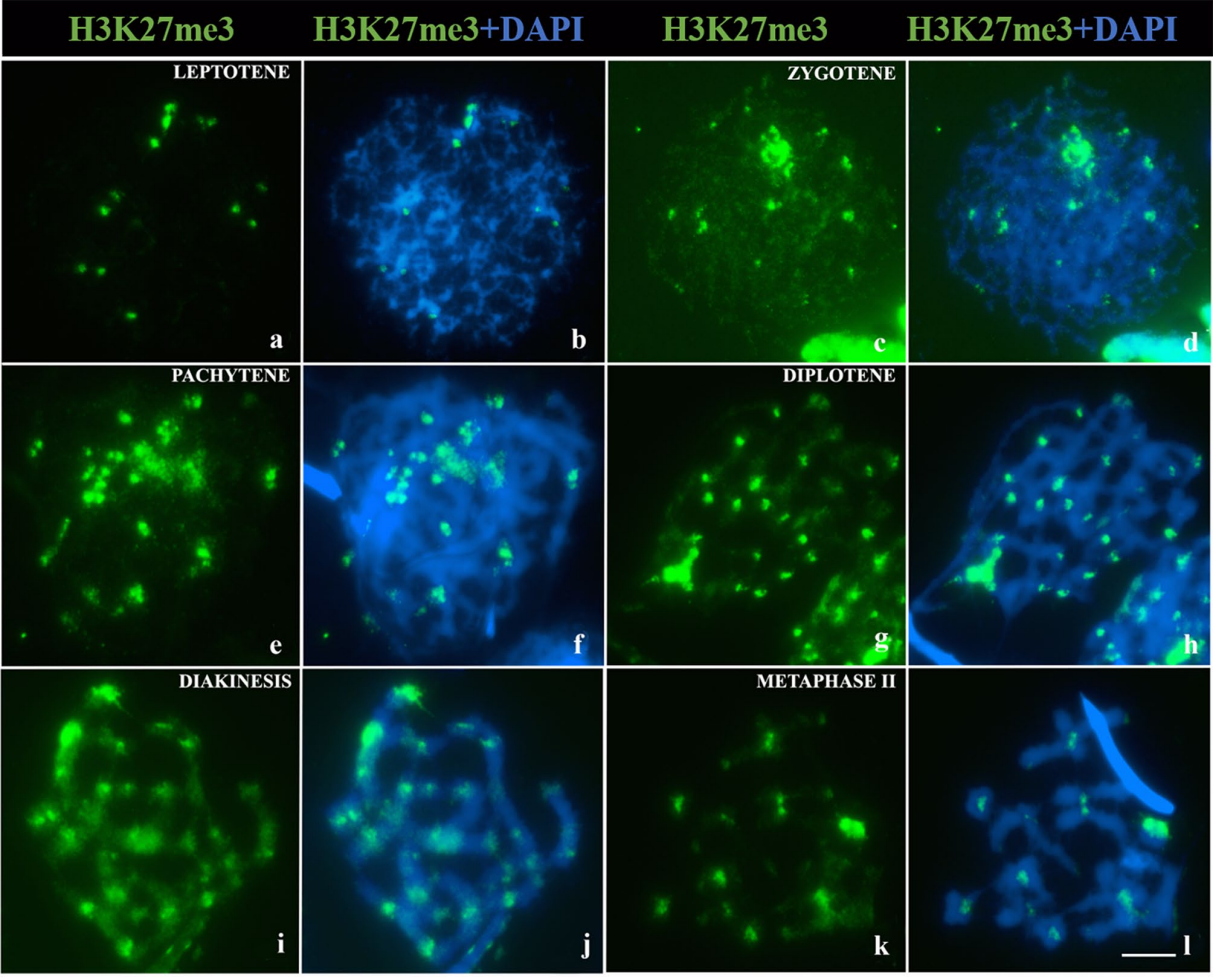
Distribution of H3K27me3 in meiosis I of *L. pentadactylus*. The H3K27me3 marking is shown in green; chromosomes were counterstained with DAPI. (**a,b**) Leptotene. (**c**,**d**) Zygotene. (**e,f**) Pachytene. (**g,h**) Diplotene. (**i,j**) Diakinesis/metaphase I. (**k,l**) Metaphase II. Barr = 10 μm.

Immunodetection of H3K27me3 was performed to investigate the role of this epigenetic modification in transcriptional inactivation of chromatin during *L. pentadactylus* prophase I. H3K27me3 was observed in isolated regions of chromatin during leptotene (Fig. 5a,b), with more intense expression in an isolated region also observed during zygotene (Fig. 5c,d). During pachytene and diplotene, this epigenetic marker was observed on the synaptic or asynaptic axis of chromosomes (Fig. 5e–h). This pattern of H3K27me3 distribution was also observed during diakinesis/metaphase I, with additional weak signals throughout other chromosomal regions (Fig. 5i,j). In metaphase II, H3K27me3 was observed only in the pericentromeric heterochromatin (Fig. 5k,l).

## Discussion

The present results confirm that 2n = 22 is conserved in the Amazon frog *L. pentadactylus*. However, the number of meiotic ring components was found to vary intraspecifically, with ten chromosomes observed in the present study and 12 in a previous study of this species^34^. These cytotypes differ in relation to a large bivalent observed in our sample. In specimens from the state of Mato Grosso, this bivalent was regarded as likely being a component of the multivalent complex^32–34^. Meiotic chains, whether associated with autosomal or sex chromosomes, are unstable and can bind other karyotype-associated elements, depending on the occurrence of new rearrangements during the evolutionary process^36^. In this way, we describe a new multiple sex chromosome system, X1Y1X2Y2X3Y3X4Y4X5Y5, in the Amazon frog *L. pentadactylus*. The cytotype described here may be distinct from those of other populations of *L. pentadactylus*. Similarly, a phylogenetic analysis based on mitochondrial (16S and COI) and nuclear (BDNF and C-myc) genes showed high genetic divergence among populations of *L. pentadactylus* in the Amazon basin, suggesting that chromosomal rearrangements could explain this large degree of differentiation of this populations^34^.

**Figure 6:**
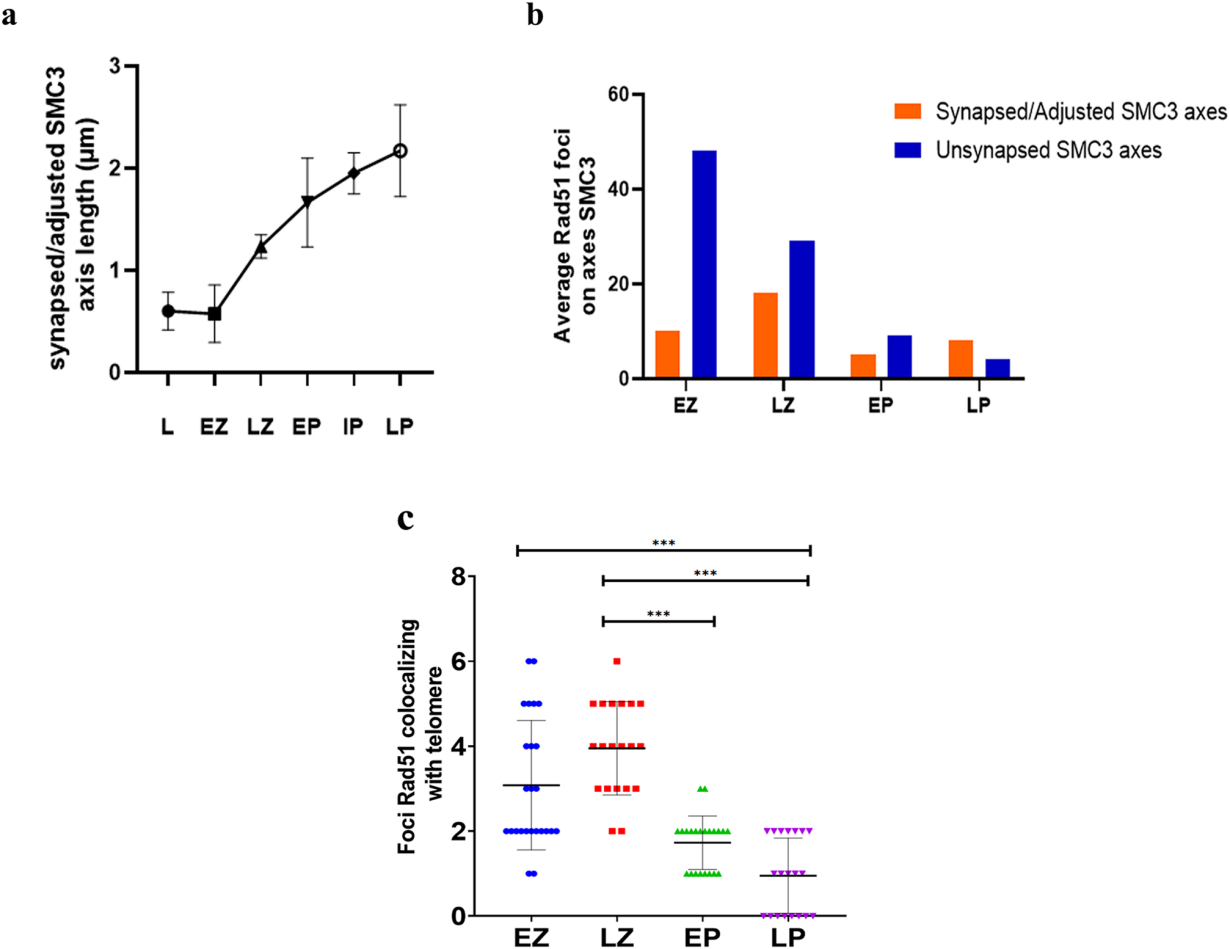
Quantitative analysis of synapsis and Rad51 foci in meiosis I of *L. pentadactylus.* (**a)** Synapsis progression during *L. pentadactylus* prophase I, based on the average of the synapsed/adjusted SMC3 axis lengths. (**b**) Distribution of Rad51 on synapsed/adjusted and asynaptic SMC3 axes; differences between the means of foci Rad51 is highly significant between zygotene and pachytene (p < 0.001). (**c**) Dot plot demonstrating the distribution of Rad51 foci in telomeric regions during *L. pentadactylus* prophase I. Each dot represents a cell (n = 88). Significant differences are observed between the means of foci of this recombinase between zygotene and pachytene (Kruskal–Wallis test, p < 0.001).

The mechanisms by which sex chromosomes pair are highly variable, with some pairings involving short terminal homologous regions of allosomes, called pseudoautosomal regions. In the platypus, for example, the sex chromosome pairing involves five pseudoautosomal regions^37^. BrdU banding indicated that the meiotic ring in *L. pentadactylus* originated from multiple translocations among terminal portions of the chromosomes involved^32^. The present study showed that these short rearranged regions are sufficiently homologous to enable meiotic pairing in these species to start regularly at chromosomal ends during zygotene. Movements of telomeres and their associations with nuclear membranes in bouquet may also contribute to the correct co-alignment of the meiotic ring components of *L. pentadactylus*^38,39^. Moreover, the presence of γH2AX and Rad51 at non-synapsed chromosomal tips in leptotene, and their subsequently becoming synapsed in zygotene (Fig. 6c), indicate that *double strand-breaks* (DSBs) during early prophase I are important for meiotic pairing in this species (see section “Introduction”). The recombinases Rad51 and Dmc1 repair these breaks using the homologous chromosome as a template^40^. In *Danio rerio,* for example, the activation of these enzymes next to telomeres is almost completely homologous for initiation of local synapses^39^. This event may be needed to recognize homologous sequences and start the organization of synaptonemal complexes in *L. pentadactylus*.

**Figure 7:**
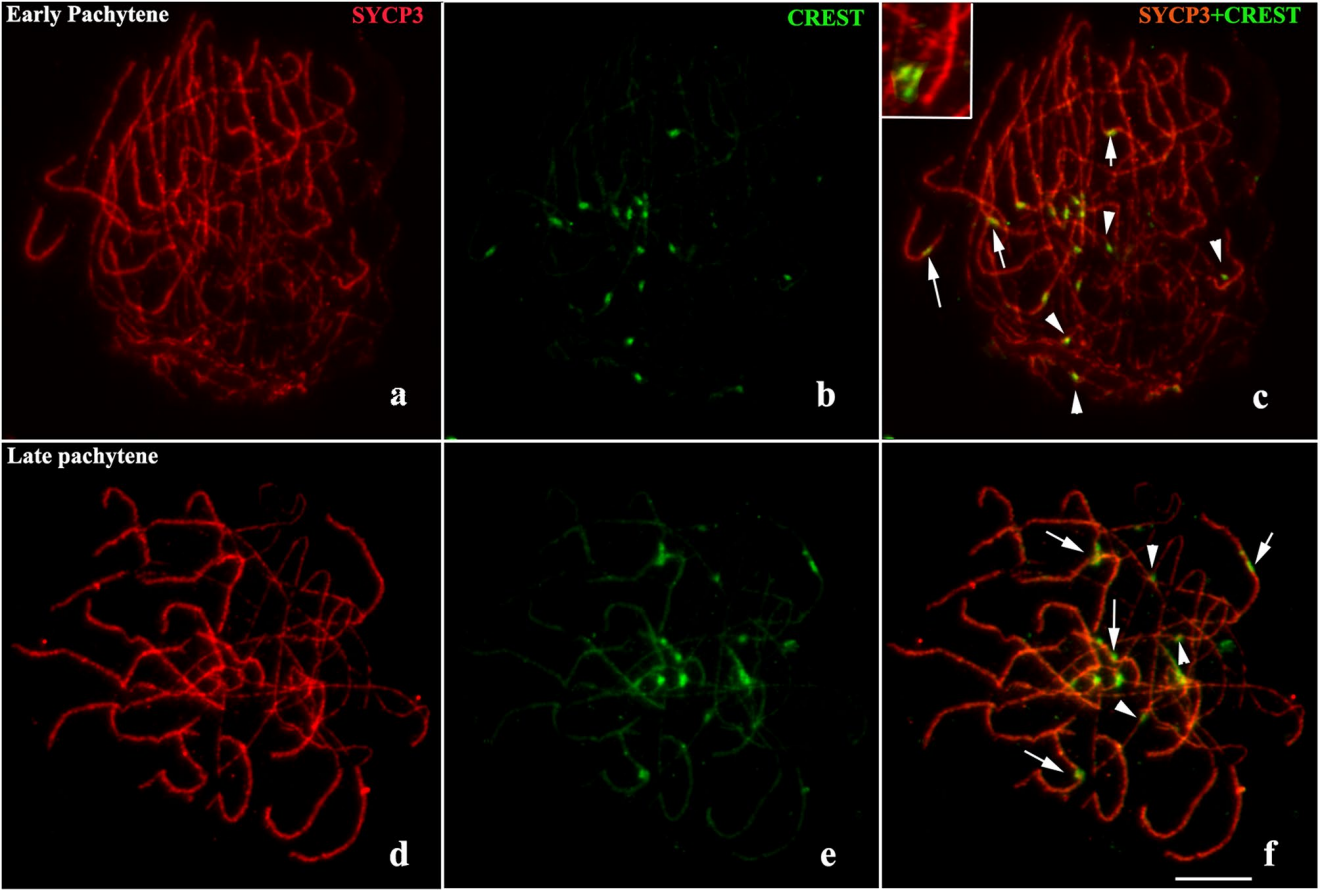
Immunodetection with SYCP3 (red) and CREST (green) showing distribution of centromeres on synapsed/adjusted and asynaptic axes of synaptonemal complex. **(a–c)** Early pachytene. **(d–f)** Late pachytene. The arrows indicate kinetochores in synapsed/adjusted regions; arrowheads indicate kinetochores in asynaptic regions. The centromere markings in asynaptic regions are highlighted in the box. Barr = 10 μm.

Once started, synapsis covers the homologous terminal portion of chromosomes, and proceeds throughout most of the length of the ring components. Because of rearrangements^32^, these non-terminal regions may lack homology, suggesting extensive heterosynapsis in this anuran species. This type of synaptic adjustment is widely found in mammals^41–43^, as it prevents the dangerous effects of transcriptional inactivation and of the action of meiotic checkpoint proteins^44^. Heterosinapses between centromeres, as observed in *L. pentadactylus* (Fig. 7), are facilitated by the fact that such regions do not need homology to perform synapse, due to their structural heterogeneity^18^. The presence of Rad51 in these heterosynapsed regions during pachytene of *L. pentadactylus* suggests that this protein can help stabilize heterosynapses after formation of the SYCP3 axis^39,43^. The multivalent center remains asynaptic until the beginning of the diplotene, suggesting that heterosynapse is not fully performed in this species. This result differs from findings in some pigs, which are characterized by complete synaptic adjustment, overtaking the breakpoint limits and not permitting central asynapsis^45^. In *L. pentadactylus*, the asynapsis in the center of the multivalent complex can also contribute to the suppression of crossing-over in this region, avoiding segregation problems during anaphase I.

The maximum degree of heterosynapsis of multivalent complexes occurs later than that of regular bivalents, which, during later pachytene, are completely synapsed or in an initial stage of disorganization of synaptonemal complexes. This asynchrony may be due to the lack of homology and by spatial arrangements of chromosomes involved in meiotic multiplexes inside the cell nucleus^46,47^. Interlocks, as observed in zygotene and pachytene of *L. pentadactylus*, are often present in individual carriers of heterozygous translocations and can delay synaptic processes^19^. Chromosomal movements associated with depolymerization and subsequent reorganization of components of synaptonemal complexes can resolve interlocks^48^. These findings suggest that some asynaptic regions and gaps registered in pachytene of *L. pentadactylus* may be associated with this mechanism of action.

At the start of meiosis I, the protein H2AX is phosphorylated by the kinase ATM, promoting structural modifications at sites of DSBs that can assist in DNA repair^49^. These findings suggest that the immunolocalization pattern of γH2AX in leptotene/zygotene of *L. pentadactylus* corresponds to regions of programmed DNA breaks, which are involved in the recombination process and contribute to homologous pairing. The results of the present study also indicate that these epigenetic modifications are highly dispersed throughout cellular chromatin during the zygotene to pachytene transition. A second wave of γH2AX formation may involve ATR kinase rather than ATM, typical of the prophase I stage^23^. Similar dynamics have been observed in eutherian mammals; however, the progression of pachytene tends to reduce the quantity of γH2AX, with the latter located only in sex chromosomes^50,51^. In contrast, γH2AX in *L. pentadactylus* persists until diplotene, albeit in reduced quantities, on all chromatins of the ring and the bivalents. The fact, that γH2AX is expressed in asynaptic regions of meiotic multiples^43,52^, may be due to the delay associated with synapsis and in DSBs repair, as they recruit silencing factors such as ATR for these regions^22^. Proto-XY has been identified in meiotic rings of *L. pentadactylus*^34^. The association of multivalents with XY sex bodies results in the spreading of γH2AX, increasing the signals of this histone variant in asynaptic regions containing the multivalents^53^. Thus, we suggest that asynaptic regions of the meiotic multivalent of *L. pentadactylus* are transcriptionally inactivated during pachytene by the formation of γH2AX.

The extensive asynapsis in *L. pentadactylus* may promote infertility in males of these species. Normally, failures in the synaptic process block the meiotic cycle, induce apoptosis, and eliminate spermatocytes, reducing the fertility of that individual^41^. Transcriptional silence is required for progression of the meiotic cycle. However, in cases of high asynapsis, spermatocytes can present errors in MSUC, inactivating genes crucial for meiosis and for the survival of spermatids, as well as initiating apoptosis^54^. Although a checkpoint in pachytene is sensitive to synapse and repair of DSBs^55^, this checkpoint can tolerate a certain degree of asynapsis^7,56^. Moreover, the stop in the meiotic cycle is dependent on the genes that are activated and inactivated because of the defect in MSUC^57^. Although the central region of the multivalent complex remains asynaptic during late/intermediate pachytene of *L. pentadactylus*, some heterosynapsed regions were positive for foci of Rad51 and γH2AX. This may be sufficient to send positive feedback to the pachytene checkpoint, allowing the normal progression of prophase I^56^. Similar results were observed in mice^58^.

Fertility analysis of *L. pentadactylus* should also consider chromosomal segregation in anaphase I. A terminal or subterminal crossing-over pattern is required for multivalents to form a zig-zag configuration during the metaphase I/anaphase I transition^36^. Only a small fraction of DSBs produced during leptotene/zygotene are repaired during cross-over^18^. The pattern of Rad51 expression during early meiosis I in the present study suggested that homologous recombination occurs in synapsed regions of the *L. pentadactylus* multivalent, adjacent to chromosomal ends, which were shown to be homologous^36^. The absence of chiasma from the interstitial regions of meiotic rings of *L. pentadactylus* is consistent with the terminal recombination observed in this amphibian species^32^. Moreover, the alternating anaphasic segregation of multivalent components of *L. pentadactylus*^32^, indicates that the meiotic cycle follows regularly, ensuring the formation of balanced gametes and contributing to the maintenance of fertility in this species.

The distribution of H3K27me3 in metaphase II observed in the present study suggested that this histone modification is an indicator of pericentromeric heterochromatin in this species. Moreover, this is the first study to show the presence of an epigenetic marker in the centromere of an anuran. In other species, H3K27me3 is generally related to facultative heterochromatin, promoting the reversible inactivation of (i) genes presents on the X chromosome^59^; (ii) repetitive DNA associated with synaptonemal complex organization^60,61^; (iii) the non-synapsed autosomal region ^24^; and (iv) the ZW chromatin of birds^26^. DSBs tend not to form in repetitive regions, as they may promote crossing-over in non-allelic regions, generating genomic instability, harmful rearrangements to chromosomal segregation^62,63^. In most organisms, DNA sequences located in pericentromeric regions are generally silenced by H3K9me3^62,64^. The simultaneous occurrence of H3K27me3 and other epigenetic markers at some genomic loci results in the simultaneous regulation of several genetic functions, including the inactivation of transposable elements^65–67^. Moreover, despite the absence of H3K9me3, the formation and stability of pericentromeric heterochromatin are maintained due to allocation of H3K27me3 to this region^68^. These findings suggest that the presence of H3K27me3 in the pericentromere during meiosis of *L. pentadactylus* may be necessary to avoid recombination adjacent to the centromere, preventing problems with chromosomal segregation, especially of elements involved in sex ring.

The present study demonstrated that the multiple sex chromosome system in *L. pentadactylus* can present interpopulation variation and suggest that a set of meiotic mechanisms intrinsic to this species (including partial synaptic adjustment, pericentromeric sequence inactivation by H3K27me3, MSUC and persistence of Rad51 foci) help regulate the meiotic cycle and ensure the maintenance of fertility in this anuran. These findings demonstrate the high functional plasticity of meiotic proteins, and allow us to understand the way in which cells of the germ line in a primitive tetrapod adapt to the wide occurrence of autosomal-sexual translocations, allowing their fixation in the population.

## Methods

### Sample

The analyzed sample was composed of three *L. pentadactylus* males in the municipalities Abaetetuba and Canaã dos Carajás, Pará, Brazil. All institutional and national guidelines for the care and use of laboratory animals were followed. This research was approved by the “Comitê de Ética em Pesquisa com animais de experimentação” (Ethics Committee in Research with experimental animals) from the Universidade Federal do Pará, reference number 68-2015. JCP has a permanent field permit, number 13248 from “Instituto Chico Mendes de Conservação da Biodiversidade”. The Cytogenetics Laboratory from UFPa has a special permit number 19/2003 from the Ministry of Environment for samples transport and 52/2003 for using the samples for research. These specimens were deposited in the collection of Laboratório de Citogenética da UFPA.

### Synaptonemal complexes

Testes were kept in Hanks buffered saline solution for 10 min, and hypotonized in 500 µM sucrose for 20 min at room temperature. These testes were subsequently macerated in 100 µM sucrose, to generate cellular suspension. About 60 µL of each solution was spread onto slides previously coated with 2% paraformaldehyde. The slides were kept in a humidified chamber for 2 h, washed in 0.4% Kodak Photo-flo solution, and stored at – 80 ºC.

### Antibodies

The primary antibodies utilized for immunolocalization of proteins included rabbit antibody to structural maintenance of chromosomes protein 3 (SMC3) (Abcam, ab9263, diluted 1:200 in PBST); rabbit antibody to histone H2A phosphorylated at serine 139 (γH2AX) (Abcam, ab2893, diluted 1:50); rabbit antibody to the DNA repair protein RAD51 homolog 1 (Rad51) (Santa Cruz Biotechnology, H92 sc-8349, diluted 1:50); rabbit antibody to histone H3 trimethylated at lysine 27 (H3K27me3) (Cell Signal, 9733S, diluted 1:50); a rabbit anti-SYCP3 (ab15093,Abcam Ltd., UK diluted 1:100) and human CREST serum at (Laboratorios IFI, Buenos Aires, Argentina diluted 1:100).

### Immunodetection

Slides were washed in PBS, and blocked in 5% BSA, 0.1% Tween 20, for 30 min at room temperature. The slides were washed and incubated with primary antibodies for 1 h at 37 ºC in a humid chamber. After washing, the slides were incubated for 2 h at 37ºC with rabbit anti-IgG conjugated to TRITC or FITC and diluted 1:100 in PBST. The slides were again washed in 1X PBST and counterstained with DAPI containing antifading Vectashield.

### Telomeric probe

Genomic DNA of *L. pentadactylus* was extracted using the phenol/chloroform method (Sambrook et al. 1989), and a telomeric probe was produced by the polymerase chain reaction (PCR). Each sample contained 16.25 µL of sterile water; 100 ng of genomic DNA; 2.5 µL of 10X buffer solution; 1 µL of 50 mM MgCl_2_, 1 µL each of the forward (TTAGG_5_) and reverse (CCTAA_5_) primers, each at a concentration of 10 mM; 2 µL of DNTP mix (2 mM) and 0.25 µL of Taq polymerase. Amplified samples were electrophoresed in 1% agarose gel. Each 1 µg aliquot of PCR product was labeled by nick translation using digoxigenin-11-dUTP (Roche).

### Fluorescence in situ hybridization (FISH)

Testes were incubated in hypotonic solution, consisting of 0.075 M KCl for 30 min at 37 ºC, and fixed in Carnoy’s solution composed of methanol and glacial acetic acid (3:1). Slides containing meiotic preparations were treated with 1% pepsin for 20 min at 37 ºC and washed in 2 × SSC solution. The slides were subsequently dehydrated in 70%, 90% and 100% alcohol for 5 min each. The telomeric probe was denatured by incubation at 100 ºC and the solution added to the slides. The slides were then covered with cover slips, and the DNA was denatured by incubation at 70 ºC. The slides were subsequently washed in 2 × SSC and 4 × SSC-Tween 20 at 40 ºC, followed by incubation with FITC-conjugated antibody to digoxigenin. The samples were subsequently counterstained with DAPI containing antifading Vectashield.

### Statistical analysis

An average of 30 cells was analyzed to define each meiotic stage. Measurements of the bivalent and multivalent synapsed/adjusted region were obtained from 52 spermatocytes (between leptotene and pachytene) using the Drawid software^69^. The Rad51 foci count was performed on 88 spermatocytes (between zygotene and pachytene). Graphical and statistical analyzes were generated using the GraphPad 6.0 software. The normality of the data was verified using the Shapiro–Wilk test. Only foci present at the ends of the synapsed synaptonemal complex were considered, to determine the fraction of Rad51 colocated with telomeres, and the averages obtained were compared using the Kruskal–Wallis non-parametric test. Rad51 averages on synapsed/adjusted, and asynaptic axes were compared using the ANOVA and Turkey test. In all statistical analyzes, the level of significance was p = 0.005.

## Data Availability

All relevant data are within the paper. Data can be requested from the corresponding author.
